# Programmable Polyproteams of Tyrosine Ammonia Lyases as Cross-Linked Enzymes for Synthesizing *p*-Coumaric Acid

**DOI:** 10.3390/biom12070997

**Published:** 2022-07-18

**Authors:** Mingyu Jia, Zhiyuan Luo, Haomin Chen, Bianqin Ma, Li Qiao, Qinjie Xiao, Pengfei Zhang, Anming Wang

**Affiliations:** Key Laboratory of Organosilicon Chemistry and Material Technology, College of Material, Chemistry and Chemical Engineering, Ministry of Education, Hangzhou Normal University, No. 2318, Road Yuhangtang, Hangzhou 311121, China; mingyujia1129@foxmail.com (M.J.); luozhiyuan1021@163.com (Z.L.); chenhaomin0412@163.com (H.C.); mabianqin0415@126.com (B.M.); xiaoqinjie98@163.com (Q.X.); pfzhang@hznu.edu.cn (P.Z.)

**Keywords:** SpyTag/SpyCatcher, carrier-free immobilized enzyme, crosslinked enzyme protein, cell lysate, tyrosine ammonia lyase, bio-orthogonal system

## Abstract

Ideal immobilization with enhanced biocatalyst activity and thermostability enables natural enzymes to serve as a powerful tool to yield synthetically useful chemicals in industry. Such an enzymatic method strategy becomes easier and more convenient with the use of genetic and protein engineering. Here, we developed a covalent programmable polyproteam of tyrosine ammonia lyases (*TAL-CLEs*) by fusing SpyTag and SpyCatcher peptides into the *N*-terminal and *C*-terminal of the TAL, respectively. The resulting circular enzymes were clear after the spontaneous isopeptide bonds formed between the SpyTag and SpyCatcher. Furthermore, the catalytic performance of the *TAL-CLEs* was measured via a synthesis sample of *p*-Coumaric acid. Our *TAL-CLEs* showed excellent catalytic efficiency, with 98.31 ± 1.14% yield of the target product—which is 4.15 ± 0.08 times higher than that of traditional glutaraldehyde-mediated enzyme aggregates. They also showed over four times as much enzyme-activity as wild-type TAL does and demonstrated good reusability, and so may become a good candidate for industrial enzymes.

## 1. Introduction

Enzymes have made important contributions to the field of biocatalysis, with their high catalytic efficiency and strong specificity at room temperature, atmospheric pressure, and isothermal conditions [1]. However, the applications of natural enzymes in industry are greatly limited by fatal defects such as their isolation difficulty in reaction media (colloidal solution) and activity loss caused by their instability [2,3]. What is more, the purification of enzymes from cells is a very time-consuming and laborious process [4]. People have adopted various methods to make enzymes more favorable in biotechnology applications, and immobilization is one of them [5]. The immobilized enzyme first appeared in 1916; it was observed that the invertase enzyme could be physically adsorbed onto charcoal’s surface and retain its original catalytic activity [6]. Obviously, immobilization technology allows enzymes to separate easily and recover from the reaction system [7], in turn controlling the reaction process—not to mention improving the thermal and pH stability of the enzyme [1,8,9].

So far, immobilization technology based on physical adsorption [10] (such as entrapment [11]) and covalent attachment [12,13,14] (such as crosslinking [15]) has matured [16] and been widely applied in the fields of the pharmaceutical industry, food industry, and wastewater treatment. In fact, the ligation intensity between enzymes and their supporting carriers resulting from physical adsorption is far weaker than that from covalent attachment. Thus, a growing number of research works have leaned toward the covalent immobilization of enzymes—especially in carrier-free systems. Carrier-free immobilized enzyme(s), such as cross-linked dissolved enzymes (CLEs) and cross-linked enzyme crystals (CLECs) [17,18] provide a higher content of enzyme reactivity with higher space-time yields than carrier-bound enzymes, without taking into account the biocompatibility, toxicity, and cost of any supporting materials [19,20,21]. One prime example is glutaraldehyde mediated cross-linking [14,22,23], which is characterized by its low cost, high reactivity, and convenient operation and use. However, the random fixation that occurs in the glutaraldehyde coupling method often forms random polymorphisms that may harm the enzyme protein, leading to a decrease in enzyme activity. In recent years, researchers have found that non-standard amino acids (NSAAs) [24,25] with -N_3_ group-mediated bio-orthogonal immobilization enable the targeting of enzymes to vectors or proteins via the click reaction [26,27]. As a type of chemical ligation, this also raises fresh questions about the cost and difficulty of cross-linking, due to the need for introducing additional artificial amino acids. Conversely, Tag/Catcher systems—for example SpyTag/SpyCatcher [28,29,30], SnoopTag/SnoopCatcher [31,32], SdyTag/SdyCatcher [33] and DogTag/DogCatcher [34]-mediated bio-orthogonal covalent immobilization—overcomes this drawback by obtaining enzyme aggregates through spontaneous chemical reactions. Tag/Catcher offers unique biocompatibility and non-toxicity properties that are far superior to any organic and inorganic carriers, giving immobilized enzymes unlimited catalytic possibilities.

SpyTag and SpyCatcher are produced by cleaving the CnaB2 structural domain of the fibronectin-binding protein (FbaB) of Streptococcus pyogenes (Spy) into a 13-amino acid peptide (SpyTag) and a complementary 116-residue polypeptide (SpyCatcher) [35,36]. The irreversible covalent bond can be formed between the Asp117 of SpyTag and the Lys31 of SpyCatcher, which is very stable and can even withstand boiling treatment with sodium dodecyl sulfate (SDS) [37]. In the CnaB domain of FbaB, an isopeptide bond is spontaneously formed between the reactive lysine and reactive aspartic acid. The adjacent glutamate salt can stabilize the intermediate and catalyze the formation of the isopeptide bond [24]. The bio-orthogonal system of SpyTag and SpyCatcher has been successfully used in cell-surface labeling, enzyme adaptability to boiling [38,39], bioactive hydrogels [40], and protein assembly [41,42].

**Scheme 1:**
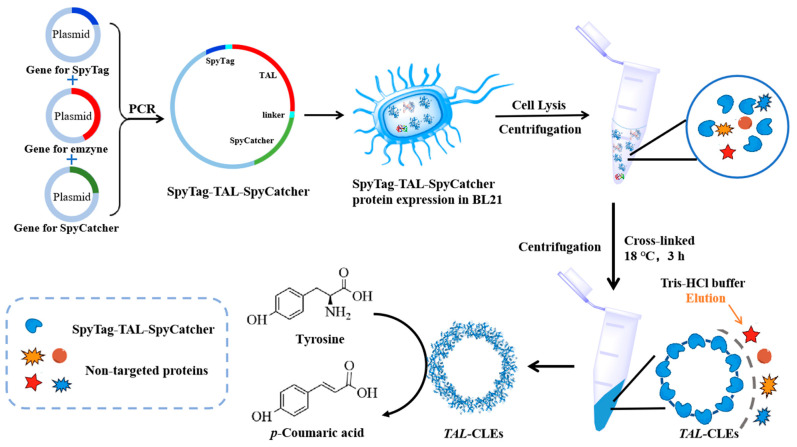
Schematic illustration of the formation of *TAL-CLEs*.

So far, research based on Tag/Catcher technic or similar isopeptide bond-initiating circularization has tended to study disposable, simple, cyclized enzymes [37,43,44]. In contrast, our group has for a long time been devoted to green biotechnology, which is more interested in converting enzymes into recyclable biocatalysts. Thus, in this work, we used a novel thermostable tyrosine ammonia lyase (TAL) from the white rot fungus *Phanerochaete chrysosporium* [45,46] as a model enzyme, and cyclized the enzyme by adding SpyTag and SpyCatcher at the *N*-terminal and *C*-terminal (Scheme 1). The specific reactions between the SpyTag and SpyCatcher are expected to form robust cross-links with a high molecular weight so as to be conveniently separated by centrifugation. The obtained SpyTag-TAL-SpyCatcher bio-orthogonally crosslinked enzymes (*TAL-CLEs*) were characterized using SEM, TEM, CLSM, and FT-IR, and the thermal stability and catalytic performance of the *TAL-CLEs* were investigated.

## 2. Materials and Methods

### 2.1. Materials

The bacteria (*E. coli* DH5*α* and *E. coli* BL21(DE3)) used in this research were ordered from Stratagene and Novagen. Plasmids pET-28a(+) were obtained from Invitrogen Corporation, Carlsbad, CA, USA. The endonuclease and protein markers were purchased from Takara Products of Hangzhou Haofeng Biotechnology Co., Ltd., Hangzhou, China. l-Arabinose was purchased from Sangon Biotech, Shanghai, China. All other chemical reagents were purchased from Shanghai Aladdin Bio-Chem Technology Co., Ltd., Shanghai, China.

### 2.2. Plasmid Construction, Protein Expression, and Characterization

#### 2.2.1. Plasmid Construction

PrimeSTAR Max DNA Polymerase, T4 DNA Ligase, and Restriction enzymes were purchased from Takara Biomedical Technology (Beijing, China) Co., Ltd. Recombinant primers were designed to carry appropriate restriction sites at 5′ and 3′ ends, and all primers used for DNA fragment amplification are listed in SI, Table S1. All the primers were synthesized by Tsingke Biotechnology Co., Ltd. The TAL gene fragment from the *Phanerochaete chrysosporium* and the SpyCatcher gene were synthesized by Shanghai Genery Co., Ltd., Shanghai, China, and their sequences are shown Table S2. The gene sequence of the constructed plasmid was examined by Tsingke Biotechnology Co., Ltd., Beijing, China.

#### 2.2.2. Protein Expression and Purification

The plasmid pET-28a(+) containing the SpyTag-TAL-SpyCatcher gene was introduced into BL21 competent cells. The engineering bacteria were pre-cultured in a 10 mL Luria-Bertani (LB) medium at 37 °C overnight. Then, a tenth of them were taken out and inoculated into 100 mL LB medium with 50 mg·L^−1^ kanamycin and cultured in a 250 mL shake flask at 37 °C, 220 rpm. When the OD_600_ reached 0.8, l-arabinose was added to a final concentration of 10 mM. When the OD_600_ reached 1.0–1.2, Isopropyl *β*-d-Thiogalactoside (IPTG) was added to a final concentration of 0.5 mM to induce gene expression. After having been cultured at 18 °C for 16 h [47], the cells were harvested by centrifugation at 8000 rpm for 5 min and resuspended in 50 mM Tris-HCl buffer (pH 8.5). Cells were lysed by ultrasound (25 KHz, 315 W, 10 min) and centrifuged at 12,000× *g* for 15 min. The bacterial lysates were immediately purified using a Ni column to obtain 1.78 g·L^−1^ SpyTag-TAL-SpyCatcher protein solution. The wild-type enzyme (WtTAL) with a His-tag on its *N*-terminal was prepared following the same steps, then stored in Tris-HCl buffer (pH 8.5). Experimental details on the purification process are detailed in the supporting information.

#### 2.2.3. Protein Characterization

SDS-PAGE was used to examine the expression of the target enzymes and verify their relative molecular weight. The sample contained a target enzyme-polyhistidine tag, which was purified by immobilized metal ion affinity chromatography with Ni-NTA agarose. Then, the purified protein solution was collected and concentrated using an ultrafiltration tube.

### 2.3. Preparation and Characterization of CLEs from the Cell Lysate

#### 2.3.1. Preparation of *TAL-CLEs* and CLEs-GA from the Cell Lysate

Crosslinked enzymes (CLEs) were prepared by spontaneous ligations of SpyTag and SpyCatcher via isopeptide-bond formation. We added the SpyTag gene to the *N*-terminus of the TAL gene and the SpyCatcher gene to the *C*-terminus via Overlap PCR. After expression of the SpyTag-TAL-SpyCatcher gene in BL21, the supernatant from the sonication treatment was condensed to 2 mL, then shaken at 220 rpm for 3 h at 18 °C to produce the desired big linkages. The aggregates were then pelleted by centrifugation at 12,000× *g* for 15 min. After centrifugation, the obtained *TAL-CLEs* was washed three times with Tris-HCl buffer (pH 8.5). We then prepared enzyme aggregates (CLEs-GA) using glutaraldehyde as the crosslinker by shaking the mixture of the WtTAL with 1.2 wt% glutaraldehyde solution for 30 min at 4 °C, 500 rpm according to the published procedure [48]. Determination of residual protein in the used supernatant was carried out by Bradford analysis. Significantly, the WtTAL could not be fully crosslinked with lower glutaraldehyde concentrations (≤0.8 wt%) and demonstrated less activity recovery (Table S3)—with abundant soluble enzymes remaining in its supernatant after being centrifuged.

#### 2.3.2. Enzymatic Activity Assay

The activity of the enzyme protein was determined by measuring the content of *p*-Coumaric acid. The reaction system for measuring the enzyme activity was 1 mL—containing 100 μL enzyme solution and 900 μL L-tyrosine (2 mmol L^−1^) reaction solution at a constant temperature of 25 °C for 1 min, after which the absorbance was measured at 310 nm. The amount of enzyme that produces 1 μmol *p*-Coumaric acid within 1 min is defined as an enzyme activity unit. The standard curve of the *p*-Coumaric acid content and OD_310_ is shown in Figure S2. It should be noted that before measuring the absorbance of the reaction solution, a 20 s centrifugation should be performed, and this time should be included into the catalytic time during which the *TAL-CLEs* were used. Otherwise, the absorbance of the solution would result in a large error in the determination of enzyme activity.

#### 2.3.3. Characterization of *TAL-CLEs*

The morphology of the *TAL-CLEs* was characterized by scanning electron microscopy (SEM, Zeiss, Oberkochen, German), transmission electron microscopy (TEM, EDAX Inc., Warrendale, PA, USA), and confocal laser scanning microscopy (CLSM, Olympus, Tokyo, Japan). To further verify the formation of *TAL-CLEs*, the WtTAL and *TAL-CLEs* were characterized by Fourier transform infrared spectroscopy (FT-IR). After Gaussian distribution fitting of the infrared results, the any changes in the secondary structure of the *TAL-CLEs* were observed.

#### 2.3.4. Thermal Stability of the *TAL-CLEs* and WtTAL

For the thermo-stability experiment, the *TAL-CLEs* and WtTAL were transferred into Tris-HCl buffer and incubated at 60 °C, 70 °C, 80 °C, and 90 °C. The prepared samples were regularly added to the tyrosine solution to detect their residual activity at 310 nm. Thermal stability refers to the residual activity of immobilized derivatives or soluble enzymes [49]. The initial activity of the enzyme preparation was 100%.

$$\mathrm{Residual}\;\mathrm{Activity}\;\left(\%\right)=\frac{Remained\;Activity}{\mathrm{Initial}\;\mathrm{Activity}\;\mathrm{of}\;\mathrm{Enzyme}\;\mathrm{Preparation}}\times 100$$

#### 2.3.5. Enzymatic Synthesis of *p*-Coumaric Acid Using *TAL-CLEs*

A total of 1 mg of *TAL-CLEs* was suspended in 1mL Tris-HCl buffer (pH 8.5), then 2 mL L-tyrosine (2 mmol L^−1^) solution was added to produce *p*-Coumaric acid. The reaction system was stirred at 37 °C. All the enzyme aggregates were separated from the reaction mixture by centrifugation after 12 h. The enzyme catalysis system was dissolved with 5 mL acetonitrile. The resulting mixture was analyzed by high performance liquid chromatography (HPLC, Agilent Technologies Inc., California, USA). The flow rate of the pump was 0.5 mL·min^−1^. The volume ratio of acetonitrile (mobile phase A) to water (mobile phase B) was 40:60. A Daicel IC column (250 mm, 4.6 mm, 5 μm particle size) was used for the chromatographic separation. The column temperature was maintained at 20 °C, and the injection volume for each injection was 5 μL. The signal acquisition wavelength was 206 nm.

## 3. Results and Discussion

### 3.1. Expression and Characterization of TAL and SpyTag-TAL-SpyCatcher

**Figure 1:**
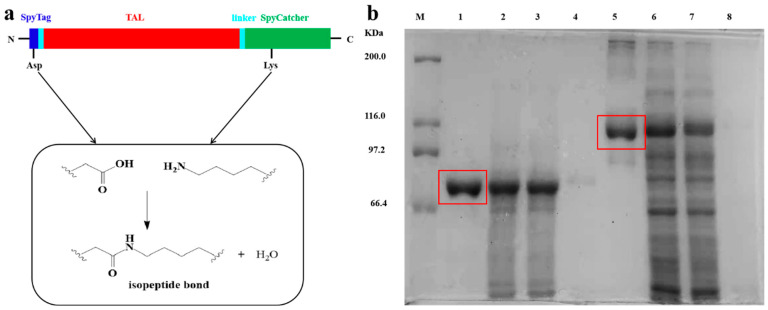
(**a**) A cartoon of the SpyTag-TAL-SpyCatcher; (**b**) SDS-PAGE analysis of enzyme proteins: Lane M, protein marker; lane 1, Purified WtTAL; lane 2, culture supernatant of WtTAL; lane 3, cellular soluble fraction of WtTAL; lane 4, cellular insoluble fraction of WtTAL; lane 5, Purified SpyTag-TAL-SpyCatcher; lane 6, culture supernatant of the SpyTag-TAL-SpyCatcher; lane 7, cellular soluble fraction of the SpyTag-TAL-SpyCatcher; lane 8, cellular insoluble fraction of the SpyTag-TAL-SpyCatcher.

The SpyCatcher-SpyTag motif derived from the CnaB2 domain of Streptococcus pyogenes mucin FbaB forms stable and specific amide bonds that provide a basis for the construction of modular proteins [50,51,52]. The CnaB2 domain is divided into two parts: one is the immunoglobulin-like domain SpyCatcher, which is composed of 138 residues (15 KDa), and the other is the short peptide SpyTag, which consists of 13 residues. We fused the SpyTag to the *N*-terminal of TAL and the SpyCatcher to the *C*-terminal (Figure 1a) via PCR technology and protein engineering. The SpyTag-TAL-SpyCatcher structure was effectively expressed in *E. coli*. In order to promote the soluble expression of TAL, the entire process of culture and induction were undertaken at a low temperature to avoid excessive accumulation of inclusion bodies. The size of the TAL protein subunit was 75 KDa and the target band in the SDS-PAGE was clearly visible, verifying the successful expression of TAL in the *E. coli* (Figure 1b, lane 1). Ideally, the molecular weight of SpyTag-TAL-SpyCatcher should be about 90 KDa. However, the actual position of the enzyme protein on the electrophoresis map was higher, indicating that SpyTag and SpyCatcher might undergo a preliminary cyclization reaction and show lower mobility (Figure 1b, lane 5) [37]. In addition, SpyTag-TAL-SpyCatcher was not fully assembled to the desired size, for it could not be detected by SEM. Accordingly, the majority of the SpyTag-TAL-SpyCatcher should be linear and contain abundant free reactive sites.

### 3.2. Morphological Characterization of *TAL-CLEs*

#### 3.2.1. SEM, TEM, and CLSM Characterization

**Figure 2:**
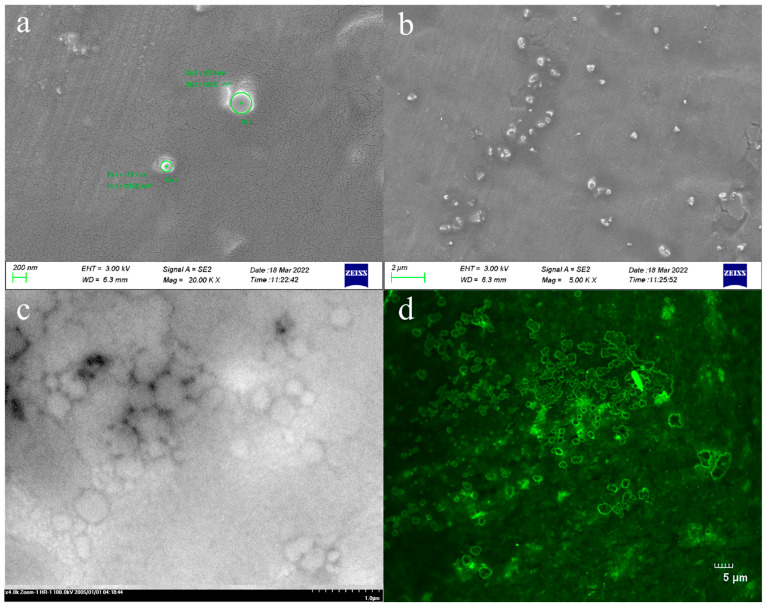
(**a**,**b**) SEM images of the *TAL-CLEs*; (**c**) TEM images of the *TAL-CLEs*; (**d**) CLSM images of the *TAL-CLEs*.

As shown in Figure 2, the prepared *TAL-CLEs* were characterized. It can be discovered from the figure that the enzyme aggregates were ring-shaped, with varying sizes. The diameter of the larger cyclic cross-linked enzymes was close to 2 μm, and some smaller cross-linked enzymes of around 200 nm were scattered around them (Figure 2a,b). We speculated that multiple SpyTag-TAL-SpyCatcher enzyme proteins may participate in intermolecular-specific responses, thus forming the circular enzyme aggregates. The result was consistent with the morphology and size observed under SEM and CLSM (Figure 2c,d), which proved the success of our method for preparing bigger cross-linked TALs to facilitate the separation of enzymes and reaction mixtures.

#### 3.2.2. FT-IR Analysis of the Secondary Structure of the *TAL-CLEs*

**Figure 3:**
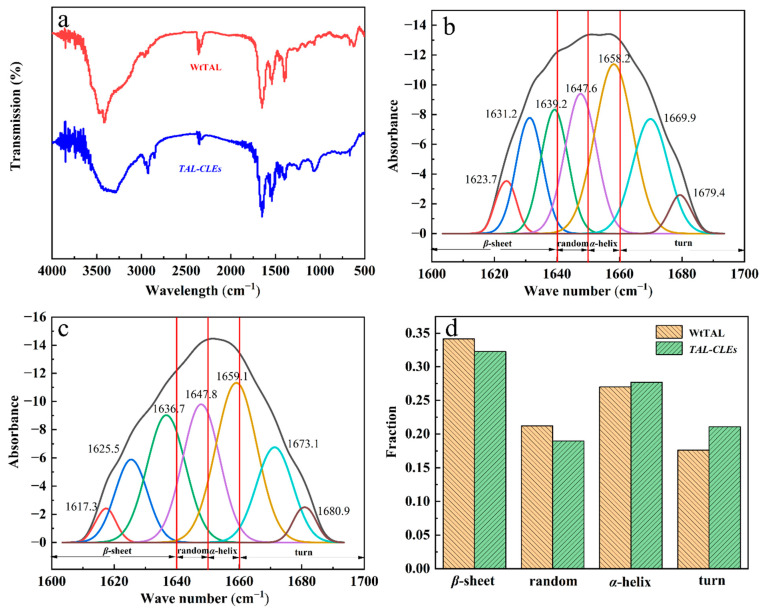
(**a**) FT-IR spectra of WtTAL and *TAL-CLEs*; (**b**) Gaussian fitting infrared spectra of *TAL-CLEs* (the residual of is 0.692, R^2^ = 0.979); (**c**) Gaussian fitting infrared spectra of WtTAL (the residual is 0.299, R^2^ = 0.990); (**d**) Comparison of peak areas of different secondary structures of WtTAL and *TAL-CLEs*.

The chemical bond types of WtTAL and *TAL-CLEs* can be observed by IR spectroscopy. It is widely known that characteristic peaks at the amide I band (1600–1700 cm^−1^) are often used to resolve the secondary structure of proteins. According to the FT-IR images, the peaks of the WtTAL and *TAL-CLEs* in the IR spectrum were basically in agreement (Figure 3a); no chemical bonds were generated or removed, so the fusion of TAL by SpyTag and SpyCatcher did not change the original chemical bond of the TAL enzyme protein. Therefore, using this SpyTag/Catcher strategy did construct carrier-free immobilized TAL proteins very well for further recycling of the TAL enzyme in the catalysis system. To further investigate the effects of crosslinking on the structure of TAL, the secondary structures of the free enzyme and *TAL-CLEs* were analyzed by Gaussian fitting in the infrared band of 1600 cm^−1^–1700 cm^−1^ using peakfit software (Figure 3b,c), and then the peak areas of the two were compared. In Figure 3d, the peak area ratios of the WtTAL and the *TAL-CLEs* with a *β*-sheet, random, *α*-helix, and turn structure were different, but at most 5%. This indicated that the *TAL-CLEs* still retained their original structure well [53]. Hence, according to the proportion in the figure, we can infer that the fusion of SpyTag and SpyCatcher does not interfere with the structure of TAL.

#### 3.2.3. Enzymatic Activity Assay

The enzyme activity of the WtTAL, SpyTag-TAL-SpyCatcher, and *TAL-CLEs* were tested. As shown in Table 1, the activity of the WtTAL and SpyTag-TAL-SpyCatcher protein were 0.43 ± 0.02 U·mg^−1^ and 0.35 ± 0.02 U·mg^−1^ (entry 1 and 2). Nearly 18.60 ± 0.82% activity loss may be attributed to the increased molecular weight of the expressed SpyTag-TAL-SpyCatcher protein, for the *N*-terminal and *C*-terminal of TAL were fused with specific polypeptides. However, the enzyme activity of the *TAL-CLEs* was significantly increased at 1.82 ± 0.03 U·mg^−1^, which was 4.23 ± 0.12-times higher than that of the wild-type TAL (entry 3). It is certain that SpyTag/SpyCatcher biotechnology has a positive effect on the modification of fragile TAL and boosts its inherent properties.

#### 3.2.4. Thermal Stability of the *TAL-CLEs*

**Figure 4:**
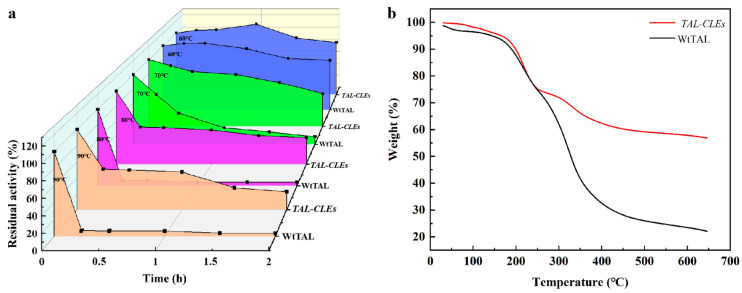
(**a**) Thermostability of WtTAL and *TAL-CLEs*; (**b**) TGA images of WtTAL and *TAL-CLEs*.

The thermostability assays of the *TAL-CLEs* and WtTAL were detected at 60 °C, 70 °C, 80 °C, and 90 °C in 50 mM Tris-HCl buffer at pH 8.5. Compared with the WtTAL, *TAL-CLEs* presented with higher stability. After incubation at 80 °C and 90 °C for 15 min, the residual activity of WtTAL was totally lost (about 7%; Figure 4a). In contrast, the designed CLEs retained 50.76 ± 1.31% and 50.38 ± 1.16% of their activity, which was 7.26 ± 0.19 and 7.52 ± 0.17-fold higher than that of WtTAL. It was not difficult to notice that the enzyme aggregates formed by the specific reactions of SpyTag and SpyCatcher increased the thermal stability of the natural enzymes. These results showed good agreement with theoretical predictions, in which cyclization can enhance protein folding [54] to improve the thermal stability of enzyme proteins. Moreover, the aggregation stability given by SpyTag/SpyCatcher cyclization might be related to the shielding of protein association in the presence of stable domains [38,55]. To gain more information about the crosslinked enzyme, we investigated the thermal degradation behavior of the WtTAL and *TAL-CLEs*. The results were clear, showing that the resistance of denaturation and degradation of the crosslinked enzyme was enhanced based on SpyTag/SpyCatcher technology, for the decomposition rate of the *TAL-CLEs* significantly slowed down above 250 °C (Figure 4b). This further proved the positive effect of the immobilization strategy on the enzymes.

### 3.3. Enzymatic Synthesis of p-Coumaric Acid Using CLEs

**Figure 5:**
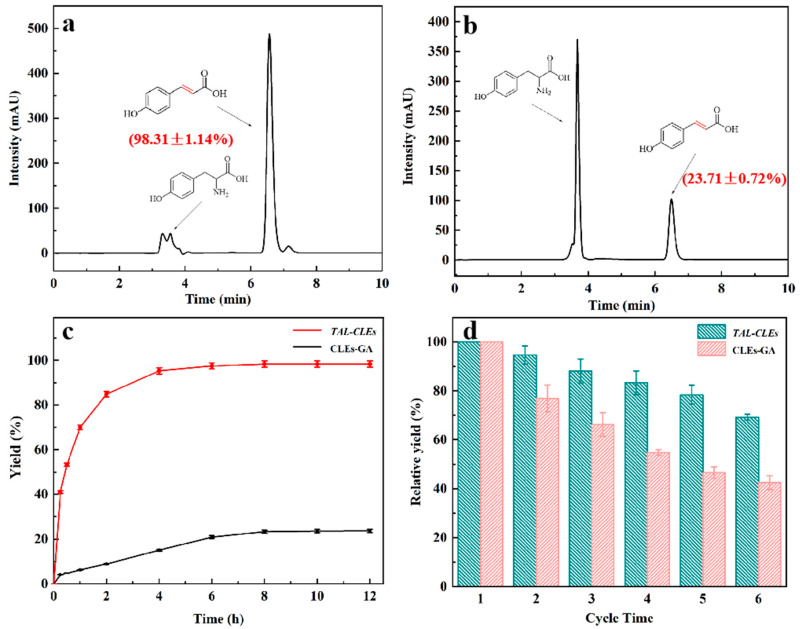
The biosynthesis of *p*-Coumaric acid using CLEs. (**a**) *TAL-CLEs*; (**b**) CLEs-GA; (**c**) The mount of *p*-Coumaric acid changed over time; (**d**) Reusability of *TAL-CLEs* and CLEs-GA.

The catalytic performance of the carrier-free immobilized enzyme was studied by the chemical reaction of tyrosine deamination to produce *p*-Coumaric acid. HPLC analysis of the standard samples of tyrosine and *p*-Coumaric acid showed that the retention times of the substrates and products were 3.66 and 6.58 min (SI, Figures S7 and S8). The amount of substrate tyrosine gradually decreased and the amount of *p*-Coumaric acid production gradually increased as the reaction proceeded. At the same enzyme content level, the *TAL-CLEs* afforded the target product a high yield of 98.31 ± 1.14%, which was 4.15 ± 0.08 times that of the CLEs-GA (23.71 ± 0.72%; Figure 5a,b). This result should be attributed to the randomness and unnecessariness of the glutaraldehyde cross-linking method damaging the enzymatic active site to some extent [49]. Moreover, exploiting genetic modifications, SpyTag-TAL-SpyCatcher-mediated immobilized enzymes could be directly prepared from cell lysates without prior purification to prevent activity loss [56,57]. The yield of *p*-Coumaric acid over time is displayed in Figure 5c. The line charts fully proved that the enzymes modified by SpyTag and SpyCatcher have excellent catalytic efficiency, which far outweighed that of other types of immobilized enzymes [58].

One of the key measures to reduce costs in industry are the reusability of immobilized enzyme catalysts [59]. Hence, we did repeat cycle experiments in order to confirm the reusability of *TAL-CLEs*. Obviously, *p*-Coumaric acid remained at 70.13 ± 1.18% of its initial yield after the *TAL-CLEs* were reused six times. By contrast, the activity of the TAL aggregates formed with glutaraldehyde as a cross-linking agent remained at around only 42.04 ± 1.82% of their initial activity (Figure 5d).

## 4. Conclusions

In this study, we generated programmable polyproteams as circular cross-linked tyrosine ammonia lyases by fusing SpyTag and SpyCatcher at the *N*-terminal and *C*-terminal, respectively. The SpyTag/SpyCatcher biotechnology proved to be extraordinarily effective in enhancing the stability of the TAL and retained its inherent secondary structure. In particular, the relative activity of the *TAL-CLEs* was 4.23 ± 0.12-times higher than that of the WtTAL. This modified enzyme also showed excellent catalytic performance in the biocatalytic synthesis of *p*-Coumaric acid—with a 98.31 ± 1.14% yield—which is a key precursor for the biosynthesis of flavonoids. Thus, this green method for the preparation of crosslinked enzymes from cell lysates is considered to be superior to the typical glutaraldehyde-mediated method.

## Figures and Tables

**Table 1:** Activity analysis of WtTAL, SpyTag-TAL-SpyCatcher, and *TAL-CLEs*.

Entry	Enzyme	Enzyme Activity (U·mg^−1^)
1	WtTAL	0.43 ± 0.02
2	SpyTag-TAL-SpyCatcher	0.35 ± 0.02
3	*TAL-CLEs*	1.82 ± 0.03

## Data Availability

All data are contained within the article.

## Supplementary Materials

The following supporting information can be downloaded at: https://www.mdpi.com/article/10.3390/biom12070997/s1, Figure S1: The domain of the SpyTag and SpyCatcher complex (PDB: 4MLI); Figure S2: Standard curve of *p*-Coumaric acid; Figure S3: Scanning Electron Microscopy (SEM) images of *TAL-CLEs*; Figure S4: Transmission Electron Microscope (TEM) images of *TAL-CLEs*; Figure S5: Confocal Laser Scanning Microscopy (CLSM) images of *TAL-CLEs*; Figure S6: FT-IR spectra of TAL and *TAL-CLEs*; Figure S7: HPLC analysis of tyrosine; Figure S8: HPLC analysis of *p*-Coumaric acid; Table S1: Primers used in this study; Table S2: Gene sequence; Table S3: Activity recovery of crosslinked enzyme aggregates using different glutaraldehyde concentrations.
