# Supplementary material for: Programmable Polyproteams of Tyrosine Ammonia Lyases as Cross-Linked Enzymes for Synthesizing p-Coumaric Acid
Source: Biomolecules. 2022 Jul 18;12(7):997. doi: 10.3390/biom12070997 (PMC9313006; doi:10.3390/biom12070997)
Supplement: Supplementary file 1 [file biomolecules-12-00997-s001.zip › biomolecules-1770802-supplementary.pdf]

Supporting Information

Programmable Polyproteams of Tyrosine Ammonia Lyases as Cross-linked Enzymes for Synthesizing *p*-Coumaric Acid

Mingyu Jia [1], Zhiyuan Luo [1], Haomin Chen [1], Bianqin Ma [1],

Li Qiao [1; *], Qinjie Xiao [1], Pengfei Zhang [1], Anming Wang [1; *]

[1]*College of Materials, Chemistry and Chemical Engineering,*

Hangzhou Normal University, Hangzhou 311121, P. R. China

Corresponding author:

Dr. Anming Wang

College of Materials, Chemistry and Chemical Engineering,

Hangzhou Normal University, Hangzhou 310036, P. R. China

Tel: +86-571-28865978

E-mail: waming@hznu.edu.cn; 11837066@zju.edu.cn

Contents

1. Materials 1

2. The structure of SpyTag-SpyCatcher 2

3. Primers used for DNA fragment 2

4. Protein expression and purification...8

5. Effect of different concentrations of glutaraldehyde on enzyme aggregation 9

6. Standard curve of *p*-Coumaric acid 10

7. SEM characterization of *TAL-CLEs* 10

8. TEM characterization of *TAL-CLEs* 11

9. CLSM characterization of *TAL-CLEs*..12

10. FT-IR characterization of WtTAL and *TAL-CLEs*........................13

11. HPLC analysis images...14

1. Materials

The strains (*E. coli* DH5α and *E. coli* BL21) and plasmid pET-28a(+) (contains a inbuilt His-tag gene) used in the study were obtained from laboratory storage. The TAL genes and PCR primers used in this study were all synthesized by Shanghai Generay Biotech Co. Ltd. The endonuclease and protein markers used were purchased from Takara Products of Hangzhou Haofeng Biotechnology Co., Ltd. The target gene SpyTag and SpyCatcher was linked by PCR. The plasmid construction results were sent to Youkang Biotechnology Co., Ltd. Antibiotics (kanamycin) were purchased from Sangon Biotech. L-Arabic sugar was purchased from Sangon Biotech. All other chemical reagents were purchased from Sinopharm Chemical Reagent Ltd. (Shanghai) unless otherwise stated. In our experiment, deionized water was used as a solvent to prepare the Tris-HCl buffer solution.

2. The structure of SpyTag-SpyCatcher

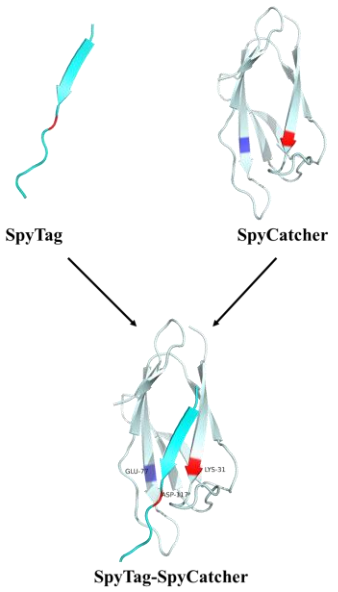

Figure S1 The domain of the SpyTag and SpyCatcher complex (PDB: 4MLI)

3. Primers used for DNA fragment

First, to obtain TAL fragment, the first round PCR was performed with TAL gene between the restriction site of NdeI and XhoI of pET-28a(+) vector as the template, and by using primers forward primer TAL F1 (5′-GGGAATTCCATATGC CGAGCCGTATTG-3′) and reverse primer TAL R1 (5′-CTCAGGGTATCAACCA TAGCTCCGCTGCCACCACTCCCTGCTTTAATGCTTTTAACCAG-3′).SpyCatcher fragment was obtained using pET-28a(+)-SpyCatcher as template and forward primer SpyCatcher F1 (5′-GCTATGGTTGATACCCTGAG-3′) and reverse primer SpyCatcher R1

(5′-CCGCTCGAGTTAAATATGTGCATCACCTTTGG-3′) as primers. In order to obtain SpyCatcher-TAL, the TAL fragment and SpyCatcher fragment obtained by the first-round PCR were used as templates, and TAL-SpyCatcher F1 (5′-GGGAATTCCATATGCCGAG-3′) and TAL-SpyCatcher R1 (5′-CCGCTCGAGTTAAATATGTG-3′) were used as primers. The PCR was then performed with the TAL-SpyCatcher fragment as the template, SpyTag-TAL-SpyCatcher F1 (5′-TGCCTATAAACCGACCAAAGGTTCAGGGGG TTCCGGTCCGAGCCGTATTGACTACT-3′) and SpyTag-TAL-SpyCatcher R1 (5′-AATATGTGCATCACCTTTGG-3′) as primers, and the PCR product was purified as the template for the next round of PCR. The primers for the next round of PCR were SpyTag-TAL-SpyCatcher F2 (5′-CATGCCATGGCACATATTGTTATGG TTGATGCCTATAAACCGACCAAAG-3′) and SpyTag-TAL-SpyCatcher R2 (5′-CCGCTCGAGTTA***GTGATGATGATGATGATG***GCTGCTGCCAATATGT GCATCACCTTTGG-3′). It is important to emphasize here that the plasmid we used, pET-28a(+), has a His tag gene between the NcoI and NdeI enzyme cut sites. The wild-type TAL gene was constructed between the NdeI and XhoI enzyme cut sites behind the His-tag gene when using pET-28a(+) as the gene vector. And to avoid the potential for unwanted effects when the His-tag is at the front of the SpyTag, we constructed the SpyTag-TAL-SpyCatcher gene inserted directly between NcoI and XhoI, and added the His-tag

(sequence in italics and bold) at the end of the gene by overlap extension PCR. We required a fragment encoding SpyTag or SpyCatcher in combination with a fragment encoding TAL (with each fragment joined via a GSGGSG linker).

Table S1 Primers used in this study

Name	Sequence (5′→3′)
TAL F1	GGGAATTCCATATGCCGAGCCGTATTG
TAL R1	CTCAGGGTATCAACCATAGCTCCGCTGCCACCA CTCCCTGCTTTAATGCTTTTAACCAG
SpyCatcher F1	GCTATGGTTGATACCCTGAG
SpyCatcher R1	CCGCTCGAGTTAAATATGTGCATCACCTTTGG
TAL- SpyCatcher F1	GGGAATTCCATATGCCGAG
TAL- SpyCatcher R1	CCGCTCGAGTTAAATATGTG
SpyTag-TAL-SpyCatcher F1	TGCCTATAAACCGACCAAAGGTTCAGGGGGTTC CGGTCCGAGCCGTATTGACTACT
SpyTag-TAL-SpyCatcher R1	AATATGTGCATCACCTTTGG
SpyTag -TAL-SpyCatcher F2	CATGCCATGGCACATATTGTTATGGTTGATGCCT ATAAACCGACCAAAG
SpyTag -TAL-SpyCatcher R2	CCGCTCGAGTTA***GTGATGATGATGATGATG***GCTG CTGCCAATATGTGCATCACCTTTGG

Table S2 Gene sequence

Gene	Sequence (5′→3′)
TAL	ATGCCGAGCCGTATTGACTACTACACCAGCAGC GGGAACGGGTACGCCCAGAGCCGTAAAAGCAG CGCCATTTATCCGGCGAGCGCCAGCACCGGGCA CGCAGCACCTAGCACCGAACGTAAACCGGAAC TGCTGGACAAATTTGTTGAAGCATACGATGAACT GCAGAGCTATCGCGAAGGAAAACCGGTTATTGT TGATGGTCACAATCTGAGCATTCCGGCCGTAGCA GCAACCGCACGTTTTGGTGCTGCAGTGGCGCTG GATGAAAATCCGGAAACACATGAACGTGTACTG CAGAGCCGTCGTGTTATTGTTGACAAAGTGAGC ACACAGCGTTCAGTTTATGGAGTTAGTACCGGTT TTGGTGGTAGCGCAGATACCCGTACCAGCGATCC GCTGCAGCTGGGTCACGCTCTGCTGCAGCATCA GCATGTTGGCGTTCTGCCGACCCAGACCGAGAG TCCGCTGCCGGCACTGCCTCTGGGTGATCCTCTG GCCACCACCTCAATGCCAGAAGCATGGGTTCGT GGTGCCATTCTGATTCGTATGAATTCACTGATTCG TGGTCATAGCGGTGTTCGTTGGGAACTGATTGAA AAAATGGGTGAACTGCTGCGTGAAAATATTACC CCGCTGGTTCCGCTGCGTGGTAGCATTAGCGCAA

	GCGGTGATCTGAGCCCGCTGAGCTATATTGCAGG<br>TACACTGATTGGTAGCCCGGCAATTCGTGTTTTT<br>GATGGTCCGGCAAGCTATGGTGCACGTCGTATTC<br>TGCCGAGCAATATTGCACTGGCAAATCATGGTGT<br>TGCACCGATTCCGCTGAGCAGCAAAGAACATCT<br>GGGTATTCTGAATGGTACAGCATTTAGCGCAAGC<br>GTTGGTGCACTGGCACTGAATGAAGCAGTTCAT<br>CTGAGCCTGCTGGCACAGGTTTGTACCGCAATG<br>GGTACAGAAGCAATGATTGGTGCAGTTGGTAGC<br>TTTGATGCATTTATTCATGATACCGCACGTCCGCA<br>TCCGGGTCAGGTTGAAGTTGCACGTAATGTTCGT<br>ACCCTGCTGGAAGATAGCCAGATGGCAGTTAAA<br>GCAGAAGATGAAGTTCATATTGCAGAAGATGAA<br>GGTGAACTGCGTCAGGATCGTTATCCGCTGCGTA<br>CCGCAGCACAGTTTCTGGGTCCGCAGATTGAAG<br>ATATTCTGAGCGCACATGAAACCGTTACCCTGGA<br>ATGTAATAGCACCACCGATAATCCGCTGATTGAT<br>GGTGAAACCGGTACAGTTCATCATGGTGGTAATT<br>TTCAGGCAATGGCAGTTACCAATGCAATGGAAA<br>AAACCCGTCTGGCAATTCATCATATTGGTAAACT<br>GCTGTTTGCACAGGCAACCGAACTGATTAATCC<br>GATGATGAATCGTGGTCTGCCGCCGAATCTGGC

AGCAACCGATCCGAGCCATAATTATTTTGCAAAA

GGTATTGATATTCATCTGGCAGCATACGTTGGTG

AACTGGGTTTTCTGGCAAGCCCGGTTAGCAGCC

ATATTCAGAGCGCAGAAATGCATAATCAGGCAG

TTAATAGCCTGGCACTGGTTAGCGCACGTTATAC

CATTAGCGCACTGGATGTTCTGAGCCTGCTGACC

GCAGCATATCTGTATGTTCTGTGTCAGGCACTGG

ATCTGCGTGCAATGCATAATGATCTGCAGAGCAG

CCTGAGCGCAATTGTTCGTGAACTGCTGCCGAA

ACATTTTCCGAGCGCAGCAAAACGTGCAGATGC

ACTGCTGCCGATTCTGGAACGTACCATTTTTCGT

GCACTGAATAGCAGCAGCAGCGCAGATTGTAAA

GCACGTATGGTTAGCGTTGCAGCAAGCACCACC

ACCCCGCTGGTTGATTTTCTGAGCGCAGATGCAG

CACTGGCAAGCGAACTGGCAAATATTACCGCAT

TTCGTACCGATCTGGCAACCCGTGCAGCAGATG

CACTGACCACCCTGCGTACCCAGTATCTGGAAG

GTGCACGTGGTGCAGCACCGGCAAGCAAATATC

TGGGTAAAACCCGTCCGGTTTATGAATTTGTTCG

TGTTACCCTGAATGTTCCGATGCATGGTCGTGAA

AATCTGCATAATTTTGAAATGGGTCCGGGTGTTG

AAGATGGTATTATTGGTAATAATATTAGCACCATT

	TATGAAGCAATTCGTGATGGTAAAATGCAGAAT GTTGTTATGCAGCTGGTTAAAAGCATTAAAGCAT AA
SpyCatcher	GCTATGGTTGATACCCTGAGCGGCCTGTCTAGCG AACAGGGTCAGAGCGGCGATATGACCATTGAAG AAGATAGTGCAACCCATATTAAATTTTCTAAACG CGATGAAGATGGCAAAGAACTGGCCGGCGCCA CTATGGAACTGCGCGATAGCTCAGGTAAAACCA TTTCAACCTGGATTAGTGATGGTCAGGTTAAAGA TTTTTATCTGTATCCGGGCAAATATACCTTTGTGG AAACCGCAGCCCCGGATGGCTATGAAGTTGCAA CCGCAATTACCTTTACCGTTAATGAACAGGGCCA GGTTACCGTTAATGGTAAAGCCACCAAAGGTGA T G C A C A T A T T
SpyTag	GCACATATTGTTATGGTTGATGCCTATAAACCGA CCAAA

4. Protein expression and purification

Formula of Luria-Bertani (LB) medium: 1 % NaCl, 1 % peptone, 0.5 % yeast extract, and 50 μg/mL Kanamycin. The cell extracts of BL21 were loaded to the Ni-NTA agarose column, which was previously equilibrated with 50

mM phosphate buffer (pH 8.5) containing 300 mM NaCl. To remove nonspecific and unbound proteins, 50 mM phosphate buffer (pH 8.5) containing 300 mM NaCl and 30 mM imidazole was used to wash the column. The protein of interest (TAL or SpyTag-TAL-SpyCatcher) was eluted with 0.1 M phosphate buffer (pH 8.5) containing 300 mM NaCl and 200 mM imidazole. The purified protein was analyzed by SDS-PAGE. Protein concentrations were measured by Bradford analysis.

5. Effect of different concentrations of glutaraldehyde on enzyme aggregation

Saturated ammonium sulfate was chosen as the aggregation agent for CLEs-GA preparation. For further tests CLEs-GA were prepared using different concentrations of glutaraldehyde (0.4 wt%, 0.8 wt%, 1.2 wt%, 1.6 wt%, 2.0 wt%, 2.4 wt%).

$$\text{Activity Recovery (\%)} = \frac{Total\ Activity\ of\ CLEs - GA}{The\ Initial\ Activity\ of\ Wt - TAL} \times 100$$

As shown in Table S3, the activity recovery in the CLEs-GA first increased with increasing glutaraldehyde concentration, presumably due to a decrease in initial enzyme leakage. The maximum recovery was found at 1.2 wt% glutaraldehyde concentration. At a glutaraldehyde concentration > 1.2 wt% the activity recovery declined again. This decrease in enzyme activity may be due to the change in the quaternary structure of the enzyme because more intensive crosslinking. Thus, we chose 1.2 wt% concentration of

glutaraldehyde to prepare CLEs-GA.

Table S3 Activity recovery of different glutaraldehyde concentrations on enzyme aggregation

Glutaraldehyde concentration	0.4 wt%	0.8 wt%	1.2 wt%	1.6 wt%	2.0 wt%	2.4 wt%
Activity recovery (%)	7.74%	10.01%	13.37%	11.35%	11.35%	5.76%

6. Standard curve of *p*-Coumaric acid

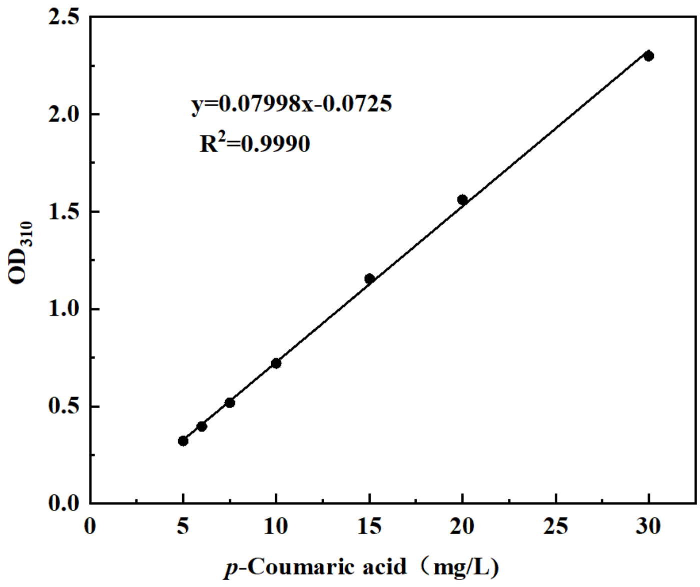

Figure S2 Standard curve of *p*-Coumaric acid

7. SEM characterization of *TAL-CLEs*

The *TAL-CLEs* were prepared by shaking at 220 rpm for 3 hours at 18 °C

and seperated by centrifugation. After drying, the samples were observed with a scanning electron microscope (JEOL-5600LV), and microscopic images were obtained under a 3.0 kV scanning electron microscope.

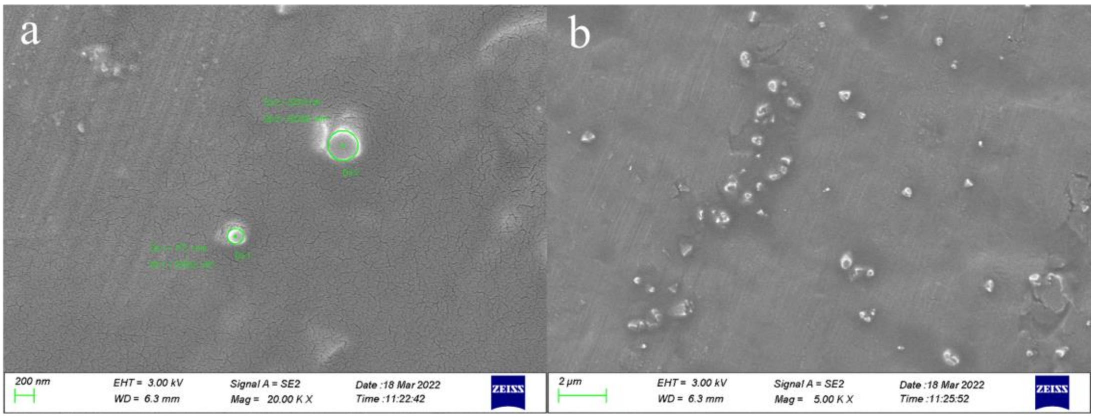

Figure S3 Scanning Electron Microscopy (SEM) images of *TAL-CLEs*

8. TEM characterization of *TAL-CLEs*

When characterizing the obtained *TAL-CLEs* using transmission electron microscope (TEM), samples were diluted to final concentrations between 0.5 and 2 nM and adsorbed on glow discharged Formvar-supported carbon-coated Cu400 TEM grids (Science Services, Munich). TEM measurements were performed on a JEOL model JEM-3010 at 100 kV.

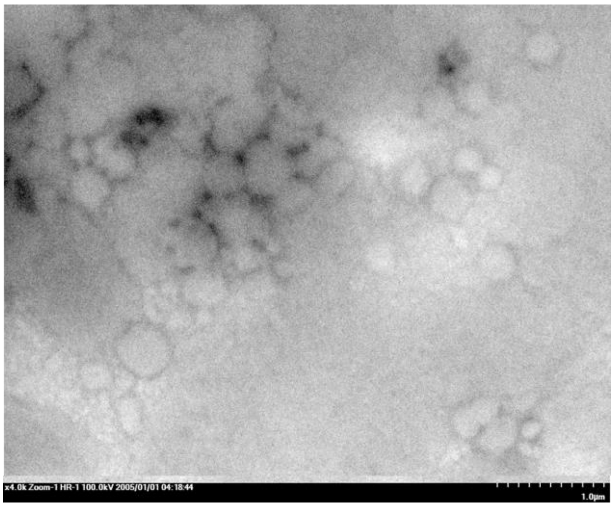

Figure S4 Transmission Electron Microscope (TEM) images of *TAL-CLEs*

9. CLSM characterization of *TAL-CLEs*

The *TAL-CLEs* were stained with fluorescent isothiocyanate (FITC) for 12 hours at 4 °C, 1 mg *TAL-CLEs* to 0.01 mg FITC. And then, the sample is washed with pH 8.0 buffer to wash excess dye, and finally a small amount of sample is dropped on the glass slide to air dry, and the cover glass is covered. The *TAL-CLEs* was observed by a laser scanning confocal microscope (Olympus FV 1000 CLSM). The enzyme protein obtained green fluorescence in the range of 480~540 nm under 405 nm excitation.

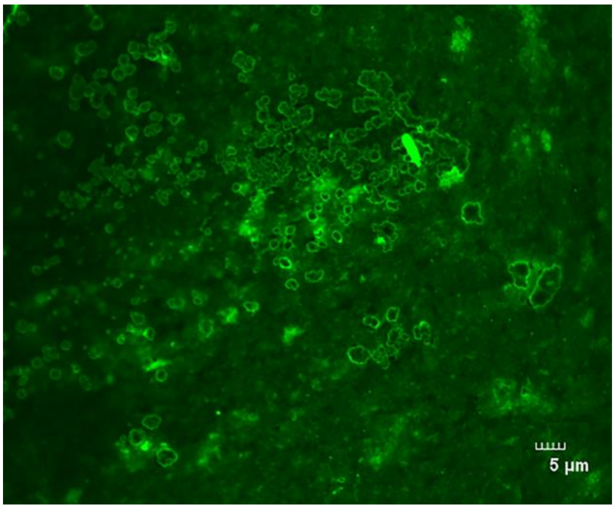

Figure S5 Confocal Laser Scanning Microscopy (CLSM) images of *TAL-CLEs*

10. FT-IR characterization of WtTAL and *TAL-CLEs*

The CLEs were prepared by shaking at 220 rpm for 3 hours at 18 °C and seperated by centrifugation. And the wild type enzymes were collected and concentrated in an ultrafiltration tube after they purified by Ni-NTA agarose. Finally, all protein samples were lyophilized and grinding mixed with KBr (protein mass : KBr mass = 1 : 100). The obtained samples were analyzed by Fourier Transform Infrared Spectrometer.

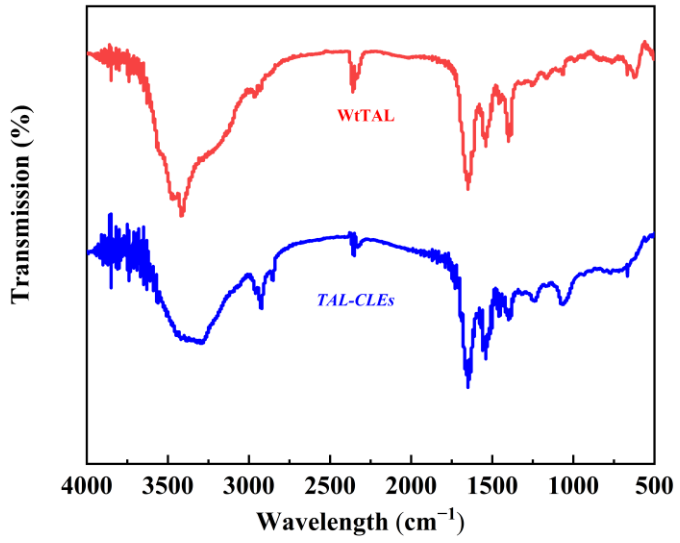

Figure S6 FT-IR spectra of TAL and *TAL-CLEs*

11. HPLC analysis images

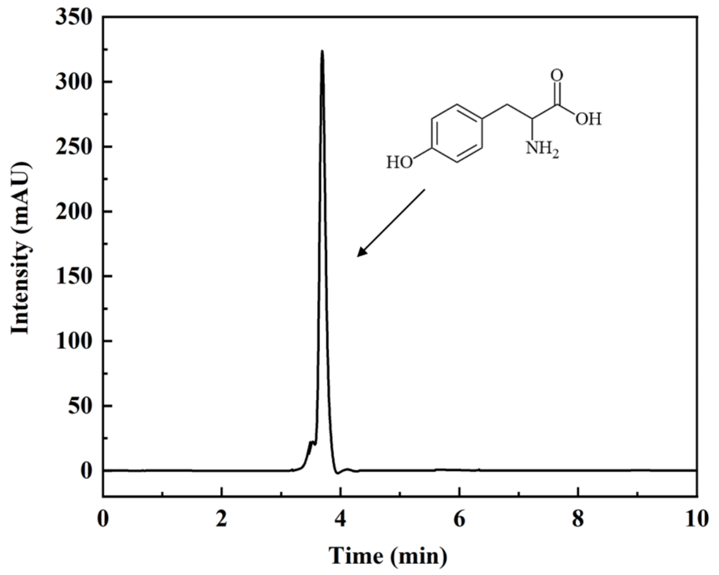

Figure S7 HPLC analysis of tyrosine.

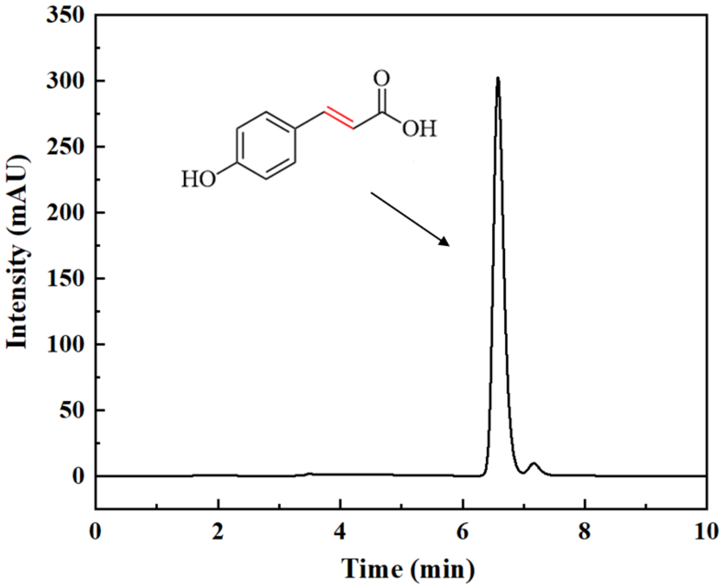

Figure S8 HPLC analysis of *p*-Coumaric acid.
